# Consumption, from a Mechanical Standpoint

**Published:** 1889-04

**Authors:** Walton Harrison


### CONSUMPTION, FROM A MECHANICAL STANDPOINT.

BY WALTON HARRISON.

The human body is a machine, because it consists of a number of inanimate elements so united, arranged and adjusted as to be capable of performing work when acted upon by a certain motive power. It is a compound machine because it is composed in part of smaller machines, the most important of which are termed organs. Each organ is required to perform a certain amount of work, to the nature of which its structure is adapted. Although the character of the task assigned to one organ may differ entirely from that assigned to another, both have a common dependence upon the general system. Each organ does its work in accordance with mechanical or physical laws, some of which are understood perfectly, others partially, and yet others not at all. The motive power is designated by the vague and general term "life," and its precise nature is no better understood by the physiologist than is the exact nature of heat or of electricity understood by the machinist.

Two organs which have assigned to them a work of great importance are the lungs. Their principal task is to remove from the system particles of worn-out flesh and other impurities brought to them by the blood, and to supply to the general system fresh oxygen from the atmosphere. The circulating system, consisting of the blood, tubes, valves and ever throbbing pump, comprises the machinery used for the purpose of transportation, and for that purpose alone.

Consumption is what might be called a "mechanical" disease. It kills not from inflammation or other incidental causes, but because it disables and renders useless a portion of the machinery. A man in the last stages of consumption dies not because his system is worn out, but because he has no lungs. With the exception that a portion of his body is virtually absent, he is otherwise a healthy man. He resembles the locomotive which would be in good running condition were it not for the fact that the slide-valves have been removed or rendered useless. He dies from the same cause which extinguishes the life of a drowning man, because his supply of air is cut off.

A consumptive becomes fearfully emaciated. The emaciation is the result, and not the cause, of the real disease. Any well-informed physician will admit that the emaciation is caused by unnatural and arbitrary decay of the lungs, no matter what may be the origin of this decay ; he will also admit the emaciation arises from a curtailment of the functionary labor of the lungs ; carrying the point further, he will admit that the emaciation results from the fact that lungs are able to handle only a limited quantity of available air. Here, then, is an important portion of the great secret unfolded. It is a law created by divine wisdom, yet so clear, simple and beautiful that when once understood it will be forever beyond the range of controversy : The emaciation incidental to consumption is simply *an effort on the part of nature to reduce the body to such a size as will be suited to the limited and constantly-decreasing capacity of the disabled lungs.* In other words when the machine, from some unknown and arbitrary cause, is rendered incompetent to perform its proper amount of work, the task is lessened in order that the work may be continued as long and as thoroughly as possible, The purpose of this article is to illustrate the truth, application and moral of this wonderful principle, which is known to everybody and recognized by none. If the principle be admitted as true, then it must also be admitted that much of the usual treatment for consumption is radically wrong, in every sense of the word ; that, should the patient recover, his recovery will be in spite of rather than through the aid of his treatment. Reference is made to the "building up" process of adding new flesh, and blood to the frail body. If a railroad engineer finds that his engine has become disabled to such an extent that it is hardly able to pull the train, he will dispose of a few cars until the necessary repairs are made : if a cannoneer discovers that his gun has become weak, he lessens the amount of his charge ; if a teamster's horse becomes lame, he will contrive a way to decrease the load ; yet an intelligent physician, when he makes the discovery that a patient's lungs have become so reduced in capacity as to be unable to do the work required by even a frail body, will endeavor to enlarge, strengthen, and if possible to fatten that body. Inasmuch as by this process the lungs themselves are not enlarged either in size or capacity, it becomes evident that they are thus made proportionately smaller and relatively weaker.

The healthy portion of the lungs will be taxed far beyond its normal capacity, for the simple reason that extra work has been added at a time when the lungs were hardly able, in fact almost unable, to accomplish the limited amount of labor which had already been assigned to them. Surely the building up of a body not supported by sufficient breathing apparatus can have no good effect, and the heavy duty and high rate of speed required of the diseased organs must inevitably hasten the untimely end.

It would be fair to answer, then, that the all-important step necessary when the disease first makes its appearance is to *assist nature in her effort.* By some legitimate means not much in conflict with the general laws of health, reduce the weight of the body a few pounds ; as a consequence, the patient will breathe freely, easily, naturally and without effort. His lungs will be relieved of much of their burden ; let his exercise and regimen, be calculated to expand and develop his lungs, rather than add flesh and weight to his general system. The patient will then, and not till then, be in condition to receive medical treatment.

